# Stride-Time Variability and Fall Risk in Persons with Multiple Sclerosis

**DOI:** 10.1155/2015/964790

**Published:** 2015-12-30

**Authors:** Yaejin Moon, Douglas A. Wajda, Robert W. Motl, Jacob J. Sosnoff

**Affiliations:** Department of Kinesiology and Community Health, University of Illinois at Urbana-Champaign, 906 S. Goodwin Avenue, Urbana, IL 61801, USA

## Abstract

Gait variability is associated with falls in clinical populations. However, gait variability's link to falls in persons with Multiple Sclerosis (PwMS) is not well established. This investigation examined the relationship between stride-time variability, fall risk, and physiological fall risk factors in PwMS. 17 PwMS (62.8 ± 7.4 years) and 17 age-matched controls (62.8 ± 5.9 years) performed the 6-minute walk test. Stride-time was assessed with accelerometers attached to the participants' shanks. Stride-time variability was measured by interstride coefficient of variation (CV) of stride-time. The participant's fall risk was measured by the short form physiological profile assessment (PPA). A Spearman correlation analysis was used to determine the relationship between variables. Increased fall risk was strongly associated with increased stride-time CV in both PwMS (*ρ* = 0.71, *p* < 0.01) and the controls (*ρ* = 0.67, *p* < 0.01). Fall risk was not correlated with average stride-time (*p* > 0.05). In PwMS, stride-time CV was related to postural sway (*ρ* = 0.74, *p* < 0.01) while in the control group, it was related to proprioception (*ρ* = 0.61, *p* < 0.01) and postural sway (*ρ* = 0.78, *p* < 0.01). Current observations suggest that gait variability is maybe more sensitive marker of fall risk than average gait parameters in PwMS. It was also noted that postural sway may be potentially targeted to modify gait variability in PwMS.

## 1. Introduction

Multiple sclerosis (MS) is a neurodegenerative disease that affects over 2 million people worldwide and an estimated 400,000 people in the United States [1]. MS is associated with inflammatory demyelination and progressive axonal damage [2]. This damage causes conduction delays in neuronal pathways and results in a myriad of symptoms including impairments in balance and gait [3].

There is increasing evidence that MS negatively impacts not only traditional spatiotemporal parameters of gait (e.g., velocity, stride length, and step time) but also the natural fluctuations observed between steps (e.g., gait variability) [4]. This observation is congruent with the view that gait variability is a unique indicator of the control of walking [5]. A recent review focusing on gait variability in MS concluded that gait variability increases early in the disease process and worsens as disability increases [4]. The review also noted a sizeable gap concerning our understanding of gait variability and MS, namely, the practical importance of gait variability [4].

One potentially important aspect of gait variability is as an indicator of falls. An association between gait variability and falls in other clinical populations has been noted [6, 7]. For instance, increased gait variability has been reported to be associated with falls in older adults and is speculated to reflect a loss of automatic rhythm of gait [8]. Although there is research documenting that gait impairment is associated with falls in PwMS [9, 10], this work has mainly focused on standard markers of gait (e.g., gait speed and stride width).

To our knowledge, there is only one study that examined the relationship between gait variability and falls in PwMS [11]. That investigation included 41 PwMS and reported that a Fourier based analysis of footfall placement variability was associated with retrospective fall status in PwMS, but standard measures of gait variability (e.g., stride-time variability) were not. Despite the novel findings, that investigation had several limitations. A major limitation of that study was that it measured gait variability over a relatively short distance (7.9 m). Previous research has suggested that gait variability metrics may not be reliable over short walking distances [12].

Another limitation is that the previous work has been descriptive in nature and not examined the contributions of physiological function to gait variability. Further understanding of modifiable physiological factors that are associated with increased gait variability could be of use in designing intervention programs to reduce gait variability and/or fall risks. In older adults, increased temporal gait variability has been linked to impaired physiological factors including impaired postural control and proprioception [13]. Although these impairments are common in PwMS, there is no data documenting similar associations between physiological domains and gait variability [14].

The current investigation was designed to investigate the relationship between gait variability and fall risk in PwMS. This study focused on stride-time variability which was previously reported as the most sensitive gait parameter to distinguish fallers from nonfallers in the geriatric population [15]. Also stride-time has been viewed as a final output of the neural system for gait control because it relies on central and peripheral inputs and feedback [16]. This primary aim of the current study was to determine whether stride-time variability during a 6-minute walk test was related to physiological fall risk in PwMS compared with controls without MS. It was hypothesized that (1) PwMS would have greater physiological fall risk, worse physiological function, and increased average stride-time and stride-time variability than the controls, (2) increased stride-time variability will be associated with greater fall risk, and (3) increased stride-time variability will be related to poorer physiological functions.

## 2. Material and Methods

All procedures were approved by the University of Illinois at Urbana-Champaign institutional review board.

### 2.1. Participants

PwMS as well as age-matched adults without neurological disease were recruited. The PwMS represented a subsample of individuals enrolled in fall prevention intervention and the control group was recruited through digital advertisements sent out to the local community. The fall prevention trial (ClinicalTrials.gov no. NCT01956227) had the following inclusion criteria: (1) a neurologist-confirmed diagnosis of multiple sclerosis; (2) ability to walk 6 minutes with or without aid; (3) self-reporting of a fall in the last 12 months; (4) age range within 50–75 years; (5) and being relapse-free for 30 days prior to assessment. The inclusion criteria for the control group were (1) being able to walk 6 minutes with or without aid, (2) having no history of neurological or orthopedic conditions that might affect their balance or mobility, (3) and meeting the age requirement (50–75 years).

### 2.2. Procedures

Upon arrival to the laboratory, the experimental procedures in detail were explained to all participants and they were provided an opportunity to ask any questions. When all questions were addressed, participants provided written informed consent. They then provided basic demographics including health history and fall history. The participants self-reported the number of falls in the previous 3 months utilizing a standardized questionnaire. A fall was defined as an event where a participant comes to rest on a lower level or the ground [17]. Participants with MS also completed the self-report expanded disability status scale (EDSS_SR_) [18]. All participants then underwent the physiological fall risk assessment and the 6-minute walk test (6MWT). The 6MWT is a validated measure of walking capacity in PwMS [19]. Use of assistive device was permitted during the testing since previous study reported that temporal gait variability in PwMS was not distinguished by usage of assistive device [14]. The participants were instructed to walk as fast as possible to cover as much distance as possible [20]. The 6MWT was conducted in a 21-meter hallway free of obstacles and distractions. The total distance walked was measured with a calibrated measurement wheel.

### 2.3. Fall Risk Assessment

Fall risk was measured utilizing the short form of the physiological profile assessment (PPA) [21]. The PPA is a standardized test that involves a series of tests including assessments of vision (visual contrast sensitivity), reaction time (simple hand reaction time), proprioception (lower limb proprioception), quadriceps strength (isometric knee extension), and postural sway on foam surface in anterior-posterior (AP) and medial-lateral (ML) axes [21]. Larger values on the proprioception, reaction time, and postural sway tests indicate worse physiological function. In contrast, smaller values on the visual contrast sensitivity and quadriceps strength tests indicate worse physiological function. The outcome of each test was combined to generate an overall fall risk score [21]. Higher fall risk scores are indicative of a person being at greater risk of falling. A score below −1 is considered a very low risk for falling, a score between 0 and 1 is a mild risk for falling, and a score of 1 and above is considered a moderate to marked risk for falling [21]. The PPA has been found to be predictive of falls in older adults [21] and PwMS [22, 23].

### 2.4. Assessment of Gait Variability

To measure the timing of the gait cycle, MTx motion trackers (Xsens Technologies B.V., Netherlands) were used. As per manufacture guidelines, sensors were placed bilaterally on the medial surface of each tibia in line with tibial tuberosity. The sensor attachment locations were optimized to reduce the skin movement artifacts [24]. Shank angle and angular acceleration in the sagittal plane were obtained at sampling frequency of 150 HZ.

**Figure 1:**
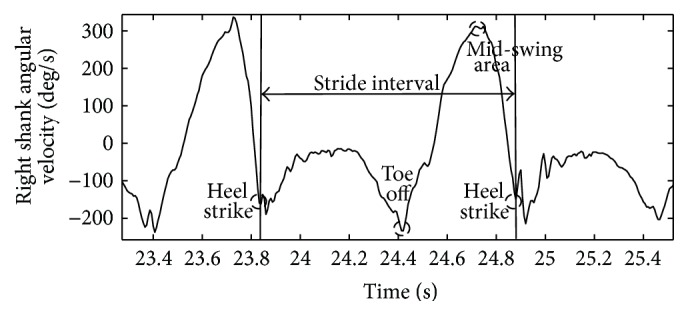
Schematic of shank angular velocity gait events and stride interval.

The stride-to-stride time interval was defined as time between consecutive heel strikes of the same foot determined using a custom MATLAB script (The MathWorks, Natick, MA, USA). Heel strike was defined as the time point where minimum negative peak angular velocity occurs immediately following the positive peak [25, 26] (see Figure 1).

Consistent with established procedures, the strides during the turns were removed [16]. To ensure that the same number of strides was analyzed across participants, the minimum number of strides produced within the sample was determined. Following this convention, all analyses utilized the first 140 strides of the 6MWT [16].

For each individual participant, the average stride-time (AVG_ST_), interstride standard deviation of stride-time (SD_ST_), and interstride coefficient of variation of stride-time (CV_ST_ = SD_ST_/AVG_ST_) were calculated. SD_ST_ is an indicator of absolute amount of stride-time variability and CV_ST_ indicates relative amount of stride-time variability [14]. In the current investigation, there was no difference in the pattern of results between SD_ST_ and CV_ST_. Therefore, for simplicity, we will report only CV_ST_. Also as there were no differences between stride-time of left and right side, we used the right side in further analyses.

### 2.5. Statistical Analysis

Statistical analysis was performed using SPSS version 21.0 (IBM, Inc., Chicago, IL, USA). Normality of outcome measures was tested using the Shapiro-Wilk test. Results were reported with average and SD for parametric parameters and median and IQR for nonparametric parameters. To examine group differences of outcomes, independent *t*-tests were conducted for normally distributed measurements while the Mann-Whitney *U* test was conducted for nonnormally distributed measurements. The magnitude of group differences was indexed by Cohen's *d* effect sizes for the independent *t*-test and by effect size *r* for Mann-Whitney *U* test. Chi-square tests were used to examine the difference of nominal parameters between groups. Due to the small sample size, spearman ranked order correlation was used to test the association between stride-time variability, fall risk, and physiological fall risk factors in each group, respectively. The significance of the difference between the two correlation coefficients was tested by Steiger's *Z*-test using Fisher's *r*-to-*z* transformation. All analyses used two-sided tests, and *p* values equal to or less than 0.05 were considered statistically significant.

## 3. Results

### 3.1. Sample Characteristics

In total, 17 individuals with MS and 17 healthy age-matched controls participated in the study. Participant characteristics including age, gender, assistive device usage, fall history, disability level, and subtype of MS and MS duration are reported in Table 1. There were no differences between the groups in gender distribution (*x* ^2^(1, *n* = 34) = 0.13, *p* = 0.71) or age (*t*(32) = 0.46, *p* = 0.65, *d* = 0.16). The MS groups had greater assistive device usage than the controls during the 6MWT. Nine out of the 17 PwMS reported two or more falls in the previous 3 months while none of the controls reported falls over the same time period.

### 3.2. Six-Minute Walk Test Performance

On average, the MS group walked 315.7 ± 84.3 meters, whereas the control group walked 570.4 ± 89.5 meters. The average gait speed of the 6MWT was 0.88 ± 0.23 m/s for the MS group and 1.58 ± 0.25 m/s for the control group. There was a significant group effect on distance and velocity (*t*(32) = 8.55, *p* < 0.01, *d* = 2.93).

### 3.3. Stride-Time

The median of AVG_ST_ of the MS group was 1.16 (IQR: 1.06–1.32) seconds whereas that of the control group was 0.95 (IQR: 0.88–0.98) seconds. The median of CV_ST_ was 3.4% (IQR: 2.9%–7.1%) for the MS group and 1.7% (IQR: 1.4%–2.2%) for the control group. Statistical analysis revealed that the MS group had significantly greater AVG_ST_ and CV_ST_ than the control group (*U* = 14, *Z* = 4.50, *p* < 0.01, *r* = 1.09; *U* = 27, *Z* = 4.74, *p* < 0.01, *r* = 1.15).

### 3.4. Physiological Fall Risk Assessment (PPA)

Detailed results of the PPA are shown in Table 2. Statistical analysis revealed that the MS group had significantly greater fall risk compared to the controls (*U* = 43, *Z* = 3.50, *p* < 0.01, *r* = 0.85). An examination of the subcomponents of the PPA revealed that the MS group had significantly worse function in all physiological factors including visual contrast sensitivity (*U* = 86.5, *Z* = 2.06, *p* = 0.05, *r* = 0.50), reaction time (*U* = 76.5, *Z* = 2.34, *p* = 0.04, *r* = 0.57), proprioception (*U* = 85.5, *Z* = 2.04, *p* = 0.05, *r* = 0.50), quadriceps strength (*U* = 86.0, *Z* = 2.02, *p* = 0.02, *r* = 0.49), postural sway in anterior-posterior axis (*U* = 79.0, *Z* = 2.26, *p* = 0.02, *r* = 0.54), and medial-lateral axis (*U* = 59.5, *Z* = 2.93, *p* < 0.01, *r* = 0.71) compared to the controls.

### 3.5. Correlation between Stride-Time and Physiological Fall Risk

**Figure 2:**
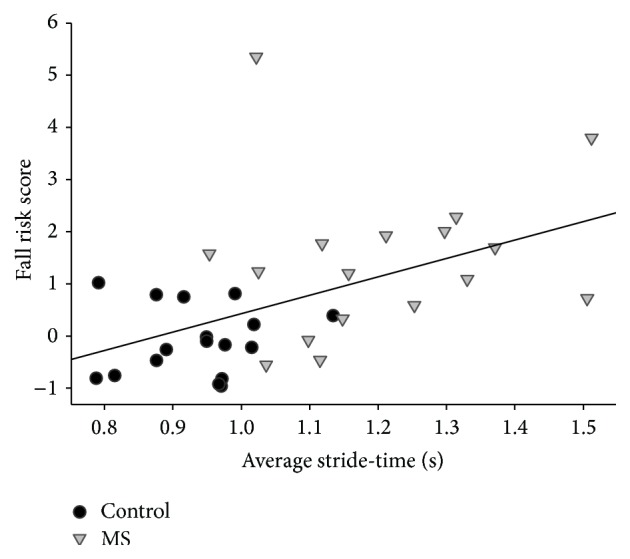
Correlation between average stride-time and fall risk in the MS and control groups.

**Figure 3:**
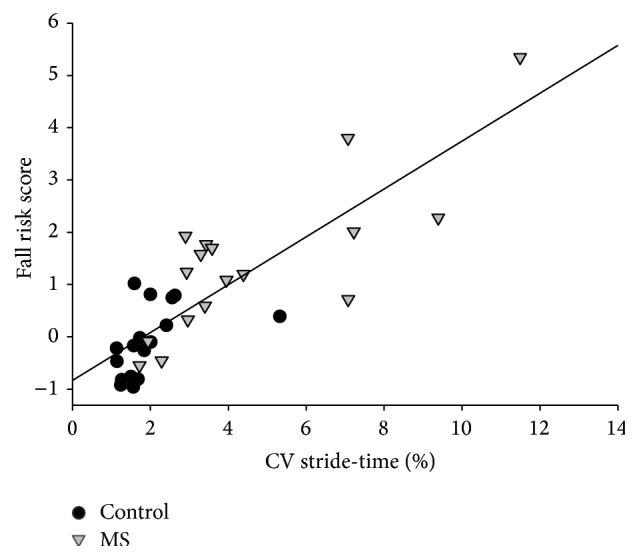
Correlation between CV of stride-time and fall risk in the MS and control groups.

Combined group analysis revealed that fall risk had a moderate positive correlation with AVG_ST_ (*ρ* = 0.55, *p* < 0.01) (Figure 2) and a strong positive correlation with CV_ST_ (*ρ* = 0.83, *p* < 0.01) (Figure 3). The association was stronger in CV_ST_ than in AVG_ST_ (*Z* = 1.51, *p* = 0.05).

Individual group correlations revealed that AVG_ST_ was not correlated with fall risk in either group (MS: *ρ* = 0.22, *p* = 0.19; controls: *ρ* = 0.06, *p* = 0.41) (Figure 2). There was a strong positive correlation between CV_ST_ and fall risk in both MS group (*ρ* = 0.71, *p* < 0.01) and the control group (*ρ* = 0.67, *p* < 0.01) (Figure 3). There was no significant difference of the strength of the correlations between the groups (*Z* = 0.2, *p* = 0.42).

An examination on correlation between gait variability and subcomponent of PPA revealed that CV_ST_ was positively correlated with postural sway along the AP (*ρ* = 0.66, *p* < 0.01) and ML (*ρ* = 0.83, *p* < 0.01) axes in the MS group. In the control group, CV_ST_ was positively correlated with proprioception (*ρ* = 0.61, *p* < 0.01) as well as sway along the AP (*ρ* = 0.53, *p* = 0.03) and ML (*ρ* = 0.68, *p* < 0.01) axes.

## 4. Discussion

The purpose of the study was (1) to examine the association between gait variability and fall risk and (2) to identify physiological factors correlating with gait variability in PwMS. Overall, it was observed that gait variability was positively correlated with physiological fall risk while average gait parameters were not in PwMS. Additionally, the results indicated that increased postural sway was related to increased gait variability in PwMS. The current findings further highlight the importance of examining the relationship between gait variability and fall risk in PwMS. Additionally, the results provide insights into which factors may be potentially targeted to modify gait variability and thus reduce fall risk in PwMS.

Consistent with previous research, PwMS had reduced walking distances compared to the controls in the 6MWT coinciding with lower gait speed [19]. Moreover, the performance on the 6MWT for the MS group is in line with norms for PwMS with moderate to severe impairment [27]. The physiological fall risk of the MS group is consistent with previous reports utilizing this prognostic test in PwMS [22, 28]. At first glance, it appears that the fall risk scores of the control group were considerably lower than that of previous reports [21]. However, the control group is considerably younger (average age of 62.6 years) than most geriatric fall research samples. The average and CV of temporal gait parameter in both groups are consistent with previous reports comparing PwMS and controls [4].

The main observation of the investigation was a significant association between the stride-time variability and physiological fall risk in both groups. It is notable that the average stride-time did not relate to fall risk in either group. Although differences in average spatiotemporal gait parameters between fallers and nonfallers in PwMS have been reported [10, 29], the predictive ability of these measures has been found to be poor [22]. The current observations are consistent with previous investigations in other populations demonstrating that the gait variability is more sensitive than standard measures of gait in predicting fall risk [30, 31]. Collectively, this raises the possibility that the stride-time variability may serve as a better predictor of falls in PwMS than average values.

The strong correlation between gait variability and fall risk was also observed in the control group. Despite the control group having significantly less gait variability and fall risk compared to the MS group, the magnitude of the relationship in the control group was congruent with that in the MS group. The finding suggests that the link between gait variability and fall risk in PwMS might not be MS dependent but rather results from a common factor between the groups such as aging related changes. In fact, the present observations are not surprising considering that the association between gait variability and falls has been reported not only in pathological populations but also in disease-free older adults [16].

In regard to physiological factors, distinctive factors related to gait variability were observed between the groups. Greater gait variability was associated only with increased postural sway in the MS group. On the other hand, in the control group, gait variability was related to both poorer proprioception and increased postural sway which was in agreement with a previous research [13]. Given the known deficits in proprioception in MS [32], it is surprising that this physiological function was not related to gait variability in MS. However, it is important to note that postural sway was more adversely impaired than proprioception in this sample. This might indicate a greater influence of postural sway on gait variability while relatively attenuating an effect of proprioception which potentially explains the absence of a relationship between proprioception and gait variability in the MS group.

Postural dysfunction in PwMS is multifaceted and it might share common pathological mechanisms that contribute to grater gait variability. Latency of conduction of sensory and motor information in PwMS could contribute to both increased postural sway and gait variability. Previous research indicated that slowed spinal somatosensory conduction and abnormal sensorimotor control are related to postural response latency leading to adverse postural control in PwMS [33]. Also, it has been speculated that an inability to adequately process incoming sensory and outgoing motor information in a timely manner may lead to inconsistent footfalls during walking, thus increasing gait variability [13]. Additionally, spasticity, the hyperexaggerated stretch reflex, might be another mediator between impaired postural control and increased gait variability. A previous study found that greater levels of spasticity at the ankle were related to impaired postural control in PwMS [34]. However, none of these factors were directly measured in this investigation.

Overall, the results suggest that gait variability may be a target of rehabilitation. In addition, it highlights that the unique underlying physiological mechanisms contributing to gait variability in PwMS should be considered when designing these clinical interventions. Previous studies demonstrated that gait and postural control can be improved through resistance and balance exercise in MS population [28, 35]. Also there is evidence that postural sway and gait variability can be reduced applying a subsensory vibratory noise to the bottom of the feet [36]. Future studies should investigate whether these interventions can be also useful in improvement of postural sway, gait variability, and/or fall risk in PwMS.

A strength of the current investigation was the use of a 21 m walkway for the 6-minute walk and analysis of 140 cycles of strides to investigate stride-time variability. The relatively long walkway and sizeable number of strides increase the probability that the spatiotemporal rhythm characteristic of walking was established by the participants [12, 37]. It is not clear that previous research documenting gait variability in PwMS was able to generate a consistent gait rhythm affecting the result [11]. Another strength of this investigation is the use of an objective prognostic metric of fall risk [21]. Indeed, previous work in this area has utilized retrospective fall recall that is suspect in populations with cognitive impairment [32].

Despite the novel observations of this current investigation, it is not without limitations. A major limitation of the study is the small sample size and relatively high level of neurological impairment in the MS group. These sample characteristics may limit the generalizability of the results and increase possibility of failure to detect subtle correlation between the factors. However, the association between gait variability and fall risk was seen in the control group, indicating that the results are somewhat generalizable. It remains to be seen whether an association between gait variability and fall risk exists in PwMS across the disability spectrum. It is possible that other factors such as medication use could influence the association between gait variability and fall risk [38]. However, medication use was not collected in the current investigation. Another limitation was that only the amount of variability was examined and consequently no information on time dependent structure of gait variability was provided. Although a data length of the current investigation was enough for investigating the amount of variability, it was insufficient for structure analysis which requires over 200 data points [39]. Fluctuations in gait have been shown to demonstrate fractal dynamics [40] and alterations of the fractal structure have been reported as a marker of gait impairment due to aging and disease [40, 41]. Therefore, further research should investigate the structure of gait variability and its relationship with fall risk in this population. Also, it is possible that variability in other spatial gait parameters (e.g., stride-length and stride-width) may yield distinct results given that other parameters imply different aspects of gait impairment [42]. It would be interesting to determine whether gait variability is a predictor of future falls in PwMS as has been seen in other populations [16].

## 5. Conclusions

In conclusion, the current observations suggest that a marker of gait variability is more strongly related to physiological fall risk than an average of gait parameter in PwMS. Future work examining whether gait variability is predictive of falls in PwMS is warranted. It was also noted that postural sway is associated with increased gait variability in PwMS. These observations highlight postural sway as a potential target of rehabilitation to modify gait variability and thus reduce fall risk in PwMS.

## Figures and Tables

**Table 1:** Participant characteristics for the MS and control groups.

	MS (*N* = 17)	Control (*N* = 17)
Age (mean ± sd)	62.8 ± 7.4 yrs	62.8 ± 5.9 yrs
Gender	11 F/6 M	12 F/5 M
Assistive device (none/cane/walker)	7/6/4	17/0/0
Number of falls in the past 3 months	2.52 ± 3.91	0 ± 0
EDSS (median (IQR))	6.0 (4.75–6.0)	—
Subtype of MS	10 RR/4 SP/3 PP	—
MS duration	19.2 ± 9.0 yrs	—

Note: F: female; M: male; RR: relapse remitting; SP: secondary progressive; PP: primary progressive.

**Table 2:** Result of physiological profile assessment of the MS and control groups.

	MS	Control	*p* value
Fall risk (*z*-score)	1.24 (0.46–1.97)	−0.17 (−0.79–0.57)	*p* < 0.01
Visual contrast sensitivity (dB)	20 (19–21)	21 (20–21.5)	*p* = 0.05
Reaction time (ms)	268.6 (239.5–268.6)	235.4 (214.7–235.4)	*p* = 0.04
Proprioception (degrees)	3.2 (1.7–5.2)	2 (1.1–3.2)	*p* = 0.05
Quadriceps strength (kg)	20.7 (15.7–26.7)	28.5 (20.7–35.9)	*p* = 0.02
Postural sway AP (mm)	23.0 (18.8–41.0)	18.0 (11.5–20.5)	*p* = 0.02
Postural sway ML (mm)	36.0 (23.0–60.0)	20.0 (11.0–22.5)	*p* < 0.01

Note: values are given in median (IQR).
